# Assessment of EEG-based functional connectivity in response to haptic delay

**DOI:** 10.3389/fnins.2022.961101

**Published:** 2022-10-18

**Authors:** Haneen Alsuradi, Wanjoo Park, Mohamad Eid

**Affiliations:** ^1^Engineering Division, New York University Abu Dhabi, Abu Dhabi, United Arab Emirates; ^2^Tandon School of Engineering, New York University, New York City, NY, United States

**Keywords:** functional connectivity, neurohaptics, haptics, PLV, graph theory, neural signal processing, EEG

## Abstract

Haptic technologies enable users to physically interact with remote or virtual environments by applying force, vibration, or motion *via* haptic interfaces. However, the delivery of timely haptic feedback remains a challenge due to the stringent computation and communication requirements associated with haptic data transfer. Haptic delay disrupts the realism of the user experience and interferes with the quality of interaction. Research efforts have been devoted to studying the neural correlates of delayed sensory stimulation to better understand and thus mitigate the impact of delay. However, little is known about the functional neural networks that process haptic delay. This paper investigates the underlying neural networks associated with processing haptic delay in passive and active haptic interactions. Nineteen participants completed a visuo-haptic task using a computer screen and a haptic device while electroencephalography (EEG) data were being recorded. A combined approach based on phase locking value (PLV) functional connectivity and graph theory was used. To assay the effects of haptic delay on functional connectivity, we evaluate a global connectivity property through the small-worldness index and a local connectivity property through the nodal strength index. Results suggest that the brain exhibits significantly different network characteristics when a haptic delay is introduced. Haptic delay caused an increased manifestation of the small-worldness index in the delta and theta bands as well as an increased nodal strength index in the middle central region. Inter-regional connectivity analysis showed that the middle central region was significantly connected to the parietal and occipital regions as a result of haptic delay. These results are expected to indicate the detection of conflicting visuo-haptic information at the middle central region and their respective resolution and integration at the parietal and occipital regions.

## 1. Introduction

Haptic technologies are becoming a vital component in human-machine communication to empower virtual and extended reality applications with multimodal and bilateral interactions (Holland et al., 2019). Telemedicine, teletraining, teleeducation, and the metaverse are a few application areas that are driving the rapid integration of haptics into human-machine interaction (Tzafestas et al., 2008). To benefit from a multimodal experience involving haptics in such applications, users should be able to experience meaningful perception of the virtual environment by simultaneously integrating inputs from all modalities (visual, auditory, and haptics). A delay in the haptic information is probable due to limited computational resources or disruption in the transmission over the network (Van Den Berg et al., 2017). Haptic delay is well-documented to cause disruption in the task completion time (Ferrell, 1965), degrade the performance (Tatematsu et al., 2011) and falsely manipulate the haptic experience (Knorlein et al., 2009).

Designing reliable and robust haptic technologies intended for use over a computer network is thus dependent on our understanding of the human experience of haptic delay. Neurohaptics is an emerging field that employs brain imaging techniques to analyze the complex neural representations provoked in response to haptic stimulation (Alsuradi et al., 2020). Functional magnetic resonance imaging (fMRI) and electroencephalography (EEG) are the two leading neuroimaging methods employed in the neurohaptics field (Alsuradi et al., 2020). However, EEG is more widespread due to its compatibility with electronic devices, lower cost, and higher temporal resolution (Alsuradi et al., 2020). The latter advantage is particularly crucial in investigating time-related perceptual attributes such as haptic delay.

Meanwhile, a few studies have been conducted to explore the neural mechanisms and activation associated with haptic delay. A study on visuo-haptic mismatch in virtual reality showed that there exists an event-related potential (ERP) based neural marker of haptic delay, namely a significant modulation of the early negativity component of the ERP detected at FCz electrode (Gehrke et al., 2019). Several other studies used time-frequency analysis to find spectral features of visual-motor incongruency; they reported that theta synchronization at the midfrontal cortex is more pronounced under incongruent cross-modal stimulation, reflecting conflict monitoring and resolution processes (Cohen and Donner, 2013; Göschl et al., 2015; Arrighi et al., 2016). We have previously shown that there exists a clear oscillatory signature of haptic delay when a discrete force feedback is delayed during passive and active interactions (Alsuradi et al., 2021). The previous work suggests an increase in the mid-frontal theta power upon the detection of haptic delay. P200 peak is also found to be modulated with the presence of haptic delay at FCz (Alsuradi et al., 2021).

However, to our knowledge, no work has investigated the functional neural networks involved in processing haptic delay. Functional connectivity refers to a group of measures that describe how spatially distant brain areas are functionally connected (Nentwich et al., 2020). Typically, functional connectivity is measured in one frequency band at a time; two brain regions are called functionally connected if either the phase or amplitude fluctuates in unison (Cohen, 2014). Neural processes associated with experiencing haptic delay are thought to be complex, and thus, examining neural activation at the electrode level could be insufficient (Lee and Hsieh, 2014). On the contrary, functional connectivity-based analysis considers the intercommunication between different brain regions, which is helpful in better understanding the underlying neural mechanisms associated with haptic delay.

The main objective of this work is to investigate the impact of haptic delay on functional neural networks and their graph theoretical characteristics. The contributions of this study are listed below:

- Examining EEG-derived connectivity patterns associated with haptic delay utilizing a hybrid approach based on phase locking value (PLV) and graph theory.
- Assessing the impact of haptic delay on global and local connectivity patterns through examining the small-worldness index and the strength index, respectively, during passive and active visuo-haptic interactions.
- Identifying the frequency bands in which significant modulation of the connectivity pattern is observed due to the experience of haptic delay.
- Locating the cortical regions(s) that act as the hub of connectivity and capturing the functional connectivity between the hub and the other brain regions during the haptic delay experience.

Toward this end, a visuo-haptic task is designed and participants are recruited to perform the task under passive and active haptic interactions. Haptic delay is introduced to evaluate its impact on the functional neural networks in action.

## 2. Materials and methods

### 2.1. Participants

Nineteen healthy subjects (9 males and 10 females; 18–40 years old) were enrolled in the study. All participants were right-handed with normal or corrected-to-normal vision. None of the participants reported a history of neurological or psychiatric disorders. Participants below the age of 18 or left-handed with reported traumatic brain injuries, neural abnormalities, or muscle atrophy were excluded from the study. The study was conducted after obtaining approval of the experimental protocol from the New York University Abu Dhabi Institutional Review Board (IRB: #HRPP-2019-120). The study was carried out in full compliance with the ethical standards outlined in the Declaration of Helsinki, following its guidelines and regulations. Before enrolling in the study, each participant signed a written consent in accordance with the IRB ethics. All participants received monetary compensation upon completion of the task.

### 2.2. Experimental setup and task

Participants were introduced to the haptic-visual task in which they used a computer screen and a haptic device (Geomagic Touch, 3D Systems, United States). A tennis ball and a racket were shown on the screen; the goal of the task was to bounce the ball using the racket, which is controlled by the stylus of the haptic device. Participants were asked to stay seated on a chair in front of the screen and were instructed to hold the stylus of the haptic device in their right hand. Participants had to perform two types of tasks, namely passive and active, under two haptic delay conditions (0 ms delay or synchronous and 220 ms delay). During the passive task, participants held the stylus passively and pressed the button on the stylus end to initiate a free fall of the ball toward the racket. During the active task, however, participants had to move the stylus actively and hit the stationary ball. In both scenarios, and upon the ball's visual collision with the racket, a discrete force feedback is delivered through the haptic device. While the haptic feedback was delivered instantly along with the visual collision in the no delay condition, it was delayed by 220 ms from the visual collision in the delay condition. The task was developed using the Unity game engine version 2018.4.5f1 (Unity Technologies, United States) and the Openhaptics Unity toolkit (3D Systems, United States).

**Figure 1:**
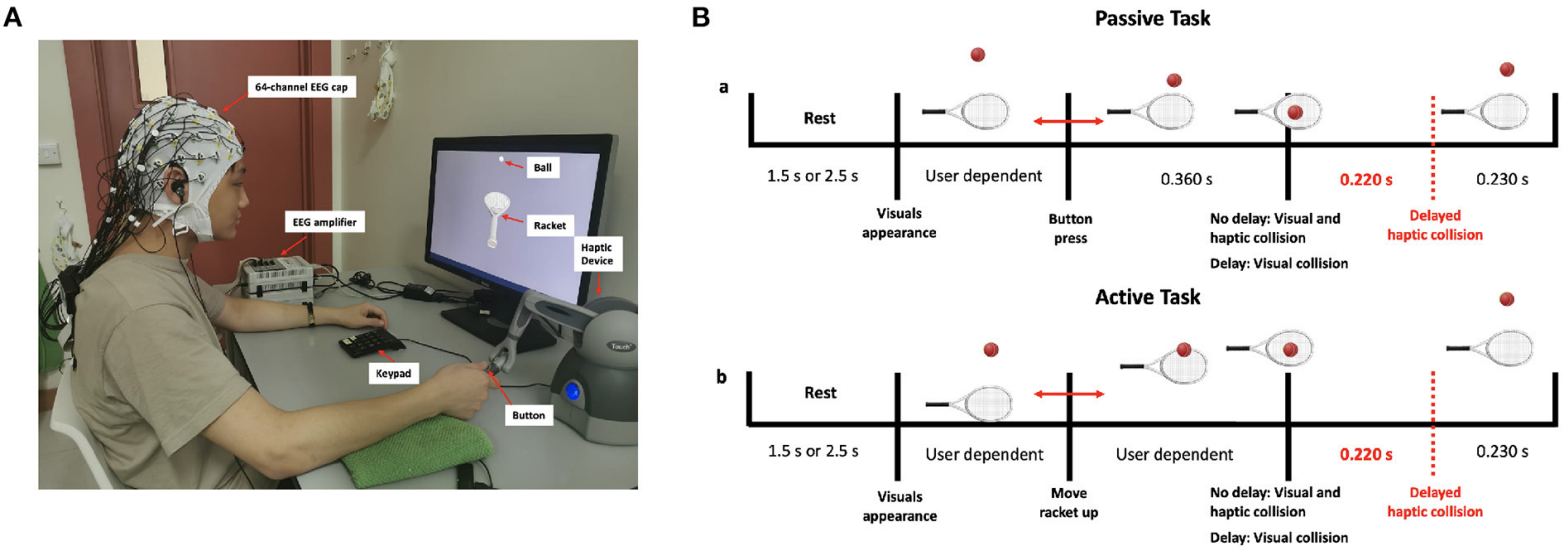
**(A)** Experimental setup showing a participant correctly holding the stylus of the haptic device **(B)** Timeline of the passive and active tasks.

Participants were trained to use the haptic device before the start of the experiment to maintain minimal body movement and thus avoid excessive motor EEG artifacts. In the active task, participants were asked to only move their wrists up or down while keeping both the elbow and the forearm rested on the table. Figure 1A shows the experimental setup, with one of the participants correctly holding the stylus of the haptic device. The timeline of a single trial for the passive and active tasks is shown in Figure 1B. The trial consists of a rest period of 1.5 or 2.5 s (randomized) during which a blank screen is displayed, followed by a single bounce move. The experimental session consisted of 10 runs (5 passive and 5 active), each comprising 20 trials. In a single run, trials were sequenced randomly and split equally between the synchronous and delay conditions. In total, each participant performed 200 trials equally divided between the four experimental conditions [Passive No Delay (PND), Passive Delay (PD), Active No Delay (AND), and Active Delay (AD)]. To maintain participants' attention and to obtain a performance measure, a question was asked at the end of 30 randomly selected trials on whether the haptic feedback was delayed. Participants had to answer using a keypad. They were also asked to fill in a post-experiment questionnaire.

### 2.3. EEG data recordings and pre-processing

EEG data were acquired from 64 active electrodes using an actiCHamp amplifier (Brainproducts GmbH, Germany) and the Brain Vision Recorder software (BVR; Version 1.21.0201 Brain Products, Germany). The locations of the electrodes were selected based on the international 10–20 system where the online reference electrode was placed at (FCz) and the ground electrode was placed at the frontal pole (Fpz). The sampling rate was 1,000 Hz. The impedance at each electrode was maintained below 10 KΩ.

EEG data were pre-processed using MATLAB release 2019b (MathWorks, United States) and the EEGLAB toolbox (v14.1.2) (Delorme and Makeig, 2004). EEG data were first filtered between 0.5 and 50 Hz using a zero-phase Hamming windowed sinc FIR filter (order: 3300) and epoched from –1 to 2 s around the visual collision event. High-amplitude artifacts, including eye blinks and muscle bursts, were tackled using the artifact subspace reconstruction (ASR) (Kothe and Jung, 2016) method. Data was then re-referenced using the Common Average Referencing (CAR) method (Lakshmi et al., 2014) and the data of the online reference channel, FCz, was restored back to the dataset.

Following re-referencing, the infomax algorithm was applied to implement independent component analysis (ICA) (Jung et al., 2000) and to maximize the statistical independence between the components of the signals. An extracted component is marked as artifactual if: (1) spurious bursts of activity are observed over the time-course of the epoch, and (2) an increase in power with an increase in frequency is observed (not a natural EEG data behavior (Buzsaki, 2006) but a common pattern of muscle artifacts' spectral power (Goncharova et al., 2003)), or (3) the topography map of the component was confined to the distant edges of the scalp, as such patterns are commonly attributed to eye or muscle artifacts. The clean components were then reflected back into the channel space. Subsequently, EEG data were spatially filtered using the current source density (CSD) method (m-constant: 4, head radius: 10 cm, smoothing constant: 10^−4^) implemented as part of the CSD toolbox in Matlab (version 1.1) (Kayser and Tenke, 2006). It is evident that applying CSD to EEG data improves the spatio-temporal features and attenuates distant effects due to volume conduction (Burle et al., 2015; Vidal et al., 2015). Spatial filtering is particularly important in studies based on channel-space functional connectivity analysis.

### 2.4. EEG data analysis

#### 2.4.1. Phase locking value

Before choosing and computing any functional connectivity measure, the pre-processed EEG data of all electrodes are band-pass filtered into five frequency bands: delta (1–4 Hz), theta (4–8 Hz), alpha (8–13 Hz), beta (13–30 Hz), and gamma (30–50 Hz). Filtered data are subsequently transformed to the time-frequency domain using Hilbert transform to obtain the phase information. The phase value is extracted for all the time-bins of each trial. Several studies emphasized the role of phase synchronization mechanisms in binding segregated brain regions (Varela et al., 2001) to fulfill several cognitive functions such as attention (Gross et al., 2004), conscious perception (Pockett et al., 2009) and memory processes (Kitzbichler et al., 2011). PLV is one of the commonly used measures in computing functional connectivity using EEG data which is intrinsically a measure of coordination across different brain regions. PLV can be used to measure the synchrony between two channels in a particular frequency band at a particular time-bin averaged over trials, forming a single *epoch*. The below equation shows the formula used to calculate the PLV index between electrodes *i* and *j* at a particular time-bin *t* (Lachaux et al., 1999; Bruña et al., 2018):

$$PLV_{i,j}\left(t\right)=\frac{1}{N}|\sum_{n=1}^{N}e^{i\left(\phi_{i}\left(t,n\right)-\phi_{j}\left(t,n\right)\right)}|$$

where *N* is the total number of trials per subject, and ϕ_*i*_(*t, n*) - ϕ_*j*_(*t, n*) is the instantaneous phase difference between channel *i* and channel *j* in trial *n* at time *t*. To reduce the computational cost, trials were divided into windows of 50 ms duration with 50% overlap, where the phase values were averaged for each window prior to calculating PLVs as described in Niso et al. (2013). A strong phase synchronization is expressed by a PLV close to 1 while a weak phase synchronization is expressed by a PLV close to 0. The choice of the baseline in this relatively complex setting was selected such that it is close to the onset (visual collision event) and consistent across conditions. The rest period is far from the onset, as can be seen from Figure 1B, while the period preceding the haptic feedback is not consistent across conditions. Thus, an appropriate choice is to use the time interval before the visual collision as a baseline. PLVs were baseline-normalized to the 200 ms data that preceded the visual collision onset. Scripts from HERMES toolbox (Niso et al., 2013) were used and adjusted for the calculation and analysis of the PLVs.

#### 2.4.2. Graph theoretical analysis

PLVs were calculated for each pair of electrodes under the five frequency bands for all participants, creating weighted undirected adjacency (i.e., connectivity) matrices of baseline-normalized PLVs. PLV matrices were analyzed using indices from graph theory (Bullmore and Sporns, 2009). In a graph theoretical analysis, the brain is modeled as a graph network of nodes and links. Generally, nodes represent brain region while links represent functional, anatomical or effective connections (Friston, 1994) depending on the neuroimaging modality as well as the calculated connectivity measure.

In this work, we consider EEG channels as nodes and PLVs as links representing functional connectivity, which generally may occur between anatomically unconnected regions. We focus on two main indices from graph theory; a global index describing the general topology of the network which is the small-worldness index (*SW*) (Muller et al., 2014) and a nodal index that describes local properties of the network which is the strength index (*S*), also called node strength (Fallani et al., 2010).

The *SW* index is defined as the ratio between the weighted average clustering coefficient (*C*) and the weighted average characteristic path length (*L*) individually normalized with respect to the frequency bands and participants. A *SW* index that is larger than 1 indicates that the network possesses small-world properties (Humphries and Gurney, 2008). A network with small-world characteristics has a high-degree of local clustering (segregation) as well as long-distance communication (integration) (Watts and Strogatz, 1998). In other words, small-worldness describes the strength of the 1) collective activations of neighbor nodes and 2) communication strength between distant nodes. The index *C* of a node is defined as the fraction of triangles around a node, where triangles are defined as simple graphs consisting of three nodes connected in the form of a triangle. The higher the number of triangles around a node, the more clustered it is. The index *L*, on the other hand, is defined as the average shortest path length between all pairs of nodes in the network. The equations below define *C*_*p, f*_ and *L*_*p, f*_ as a function of the time-bin (*t*) for a particular participant (*p*) and frequency band (*f*):

$$C_{p,f}\left(t\right)=\frac{1}{E}\sum_{j=1}^{E}C_{j}\left(t\right)$$

$$L_{p,f}\left(t\right)=\frac{1}{\left(\begin{array}{c} E\\ 2 \end{array}\right)}\sum_{i=1}^{\left(\begin{array}{c} E\\ 2 \end{array}\right)}L_{i}\left(t\right)$$

where *C*_*j*_ is the clustering coefficient for channel *j*, *E* is the number of channels, *L*_*i*_ is the characteristic path length for combination *i* of pairs of channels, and $\left(\begin{array}{c} E\\ 2 \end{array}\right)$ is the total number of channel pair combinations. Normalized *C*_*p, f*_(*t*) and *L*_*p, f*_(*t*) indices were obtained by dividing indices of the epoch to the mean of the epoch as described in Vecchio et al. (2017, 2018). The normalized *C*_*p, f*_(*t*), *L*_*p, f*_(*t*), and *SW*_*p, f*_(*t*) are defined below:

$$C_{norm}\left(t\right)=\frac{C_{p,f}\left(t\right)}{\frac{1}{T_{N}}\sum_{t=t_{0}}^{t_{n}}C_{p,f}\left(t\right)}$$

$$L_{norm}\left(t\right)=\frac{L_{p,f}\left(t\right)}{\frac{1}{T_{N}}\sum_{t=t_{0}}^{t_{n}}L_{p,f}\left(t\right)}$$

$$SW_{norm}\left(t\right)=\frac{C_{norm\left(t\right)}}{L_{norm}\left(t\right)}$$

where *C*_*norm*_ is the normalized clustering coefficient, *L*_*norm*_ is the normalized characteristic path length, *SW*_*norm*_ is the normalized small-worldenss index, *t*_0_ is the first time bin in the epoch, *t*_*n*_ is the last time bin in the epoch, and *T*_*N*_ is the total number of time bins in an epoch. Weighted indices of an epoch are obtained by averaging the indices over the epoch length to achieve a single index for every participant at each frequency band and can be realized by the equations below (Vecchio et al., 2017):

$$C_{avg-norm}=\frac{1}{T_{N}}\sum_{t=t_{0}}^{t_{n}}C_{norm}\left(t\right)$$

$$L_{avg-norm}=\frac{1}{T_{N}}\sum_{t=t_{0}}^{t_{n}}L_{norm}\left(t\right)$$

$$SW_{avg-norm}=\frac{1}{T_{N}}\sum_{t=t_{0}}^{t_{n}}SW_{norm}\left(t\right)$$

The strength index (*S*) is defined as the sum of links' weights connected to the node. The *S* index was subsequently calculated for the frequency bands that showed a statistically significant difference in the *SW*_*avg*−*norm*_ index between the synchronous and delayed conditions. The index *S* is used to identify the highly connected nodes in a specific frequency band. We used the Brain Connectivity Toolbox for the graph theoretical analysis on this work, along with custom written MATLAB routines (Rubinov and Sporns, 2010).

#### 2.4.3. Inter-regional connectivity analysis

**Figure 2:**
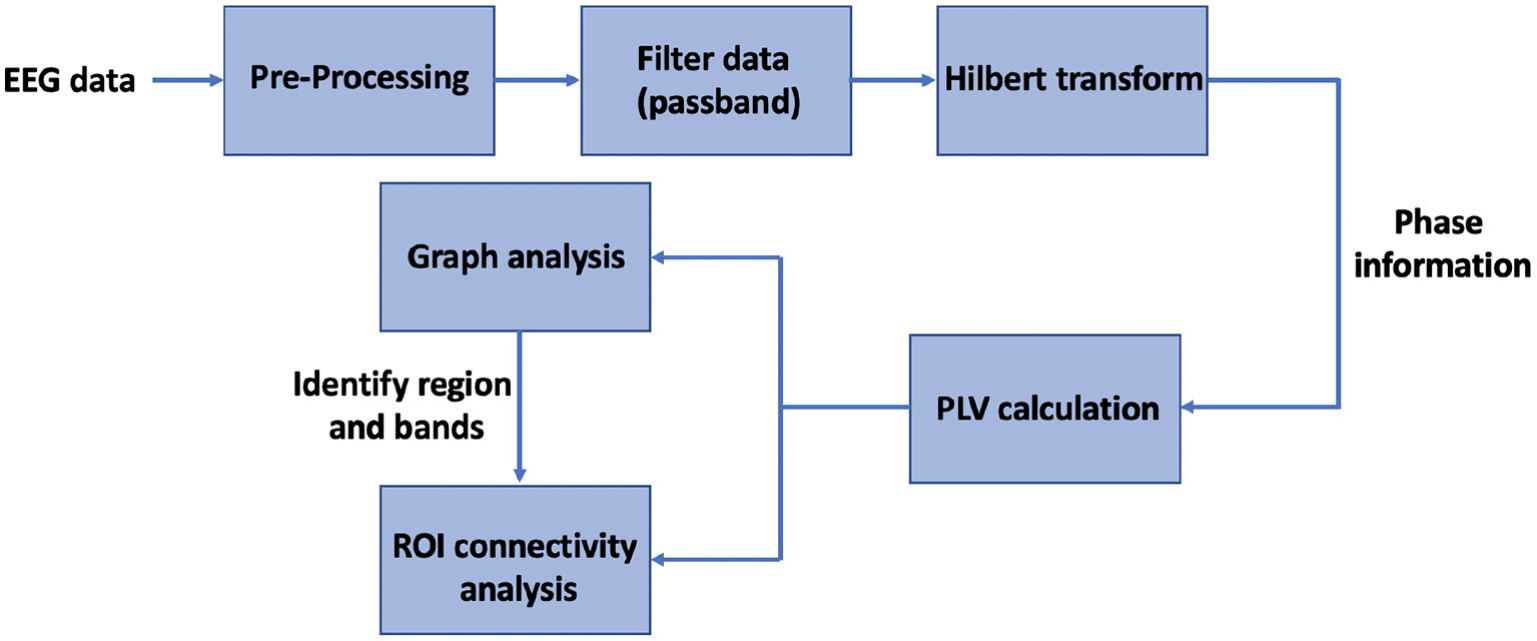
Block diagram of the sequence of steps followed for the functional connectivity analysis.

A global graph-theoretical property such as the *SW* index is useful to identify the frequency bands at which the connectivity pattern is modulated under haptic delay, while a nodal graph-theoretical property such as the *S* index is useful to locate the hubs of connectivity over the scalp. Once a hub(s) is identified, a hub-seeded connectivity analysis can be performed to capture the functional connectivity between the hub and the other brain regions. By hub seeded, we mean that we focus on evaluating the connectivity between a single electrode (the hub) and the rest of the electrodes (van Driel et al., 2012). Graph theory analysis is useful to filter out the significantly changed PLVs from the enormous adjacency matrices calculated in Section 2.4.1. Figure 2 shows a summary of the main steps taken to analyze the EEG data from a functional connectivity perspective using graph theory.

#### 2.4.4. Statistical analysis

Distributions of *SW*_*avg*−*norm*_ index were statistically tested across conditions (PND vs PD and AND vs AD) over the five frequency bands. The normality of the *SW*_*avg*−*norm*_ index distributions was tested using the Shapiro-Wilk test (Royston, 1995) implemented based on Royston R94 algorithm, which works well with sample sizes ≤ 50 (Razali and Wah, 2011; Ghasemi and Zahediasl, 2012). The test yielded an affirmation that the distributions under consideration are all Gaussian. Consequently, we used the paired *t*-test to evaluate the significant differences in *SW*_*avg*−*norm*_ index between the synchronous and delayed conditions for both the passive and active tasks across the delta, theta, alpha, beta, and gamma bands. False discovery rate (FDR) method was used to correct for multiple comparisons across the five frequency bands by adjusting the raw *p*-values; we report the adjusted *p*-values in the manuscript.

Frequency bands that showed significant differences (*p* < 0.05) were further analyzed using the *S* index. The *S* index which was calculated over the time-course of the epoch, was compared across the synchronous and delayed conditions. The statistical comparison of the *S* index was computed for the hub(s) of connectivity at the peak values of the index. This is motivated by the temporal difference of the peak values caused by haptic delay. Lastly, hub-seeded connectivity analysis is conducted. The time-course of the difference topography maps for the hub-seeded PLVs are obtained per condition. Channels that show significantly different connectivity between the synchronous and delay conditions are highlighted at every time bin. To limit type 1 error due to multiple comparisons of the 64 channels and 10 time bins, *p*-values were corrected using the FDR method. For all analyzes in the manuscript, the statistics are computed at the group level and the reported *p*-values are the adjusted values after considering the multiple comparison factor.

## 3. Results

### 3.1. Global connectivity properties

**Figure 3:**
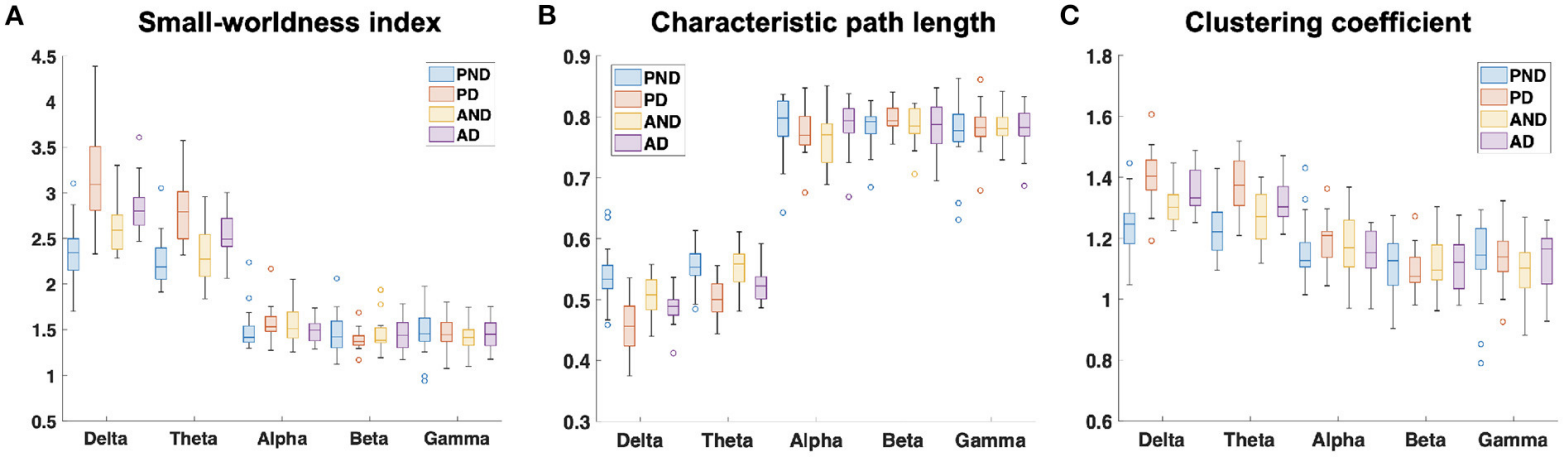
Normalized average **(A)** small-worldness (*SW*_*avg*−*norm*_), **(B)** clustering coefficient (*C*_*avg*−*norm*_), and **(C)** characteristic path length (*L*_*avg*−*norm*_) indices over the five frequency bands. PND and PD refer to (passive, no delay) and (passive, delay) while AND and AD refer to (active, no delay) and (active, delay) conditions, respectively.

**Figure 4:**
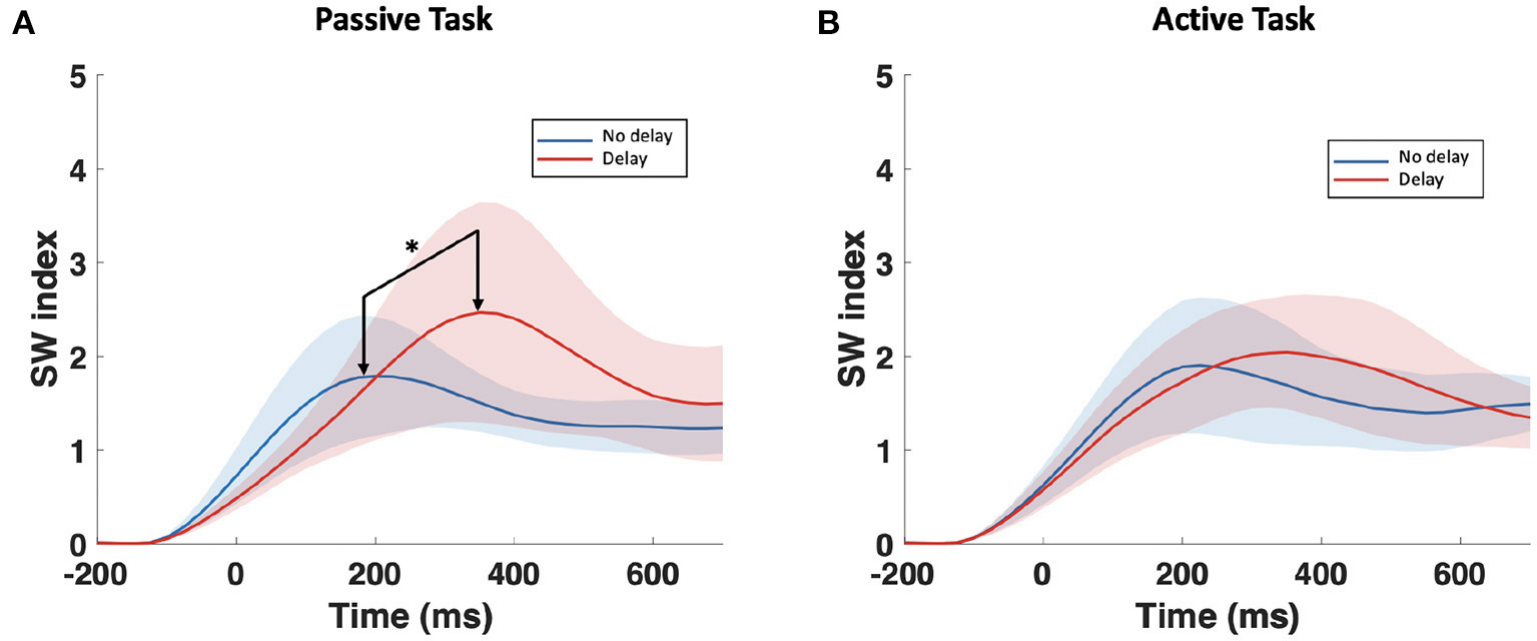
Time-course of *SW*_*norm*_ index in the delta-theta band for the **(A)** passive task and the **(B)** active task. The onset (0 ms) is the visual collision event. *Indicates statistically significant difference.

The examination of the *SW*_*avg*−*norm*_ index revealed a statistically significant difference between the synchronous and delayed conditions for the passive and active tasks (PD and AD) only in the delta and theta bands. Figure 3A shows a clear increase in the *SW*_*avg*−*norm*_ index when the haptic delay is introduced. On the contrary, the *SW*_*avg*−*norm*_ index showed no significant change upon the introduction of the haptic delay in the alpha, beta, or gamma bands. To better understand whether the change in the *SW*_*avg*−*norm*_ index is motivated by an increase in the *C*_*avg*−*norm*_ index or a decrease in the *L*_*avg*−*norm*_ index, both indices were examined. The details of the statistical analysis results on the *SW*_*avg*−*norm*_, *C*_*avg*−*norm*_ and *L*_*avg*−*norm*_ indices are found in Table 1. The time-course of the *SW*_*norm*_ index is further examined over the delta-theta bands and plotted in Figure 4 for both the passive and active tasks. Analysis shows that the peak value is significantly greater for the PD compared to the PND condition (Passive: *p*-value < 0.05, *t*_*PND*_=[175 ms] and *t*_*PD*_ = [350 ms], paired *t*-test). However, no significant difference was found over the peaks for the active task (Active: *p*-value > 0.05, *t*_*AND*_=[225 ms] and *t*_*AD*_ = [350 ms], paired *t*-test). As per Figure 3A, it is evident that the *SW*_*avg*−*norm*_ was higher on average for AD compared to the AND condition, but not over the peak values.

**Table 1:** Paired *t*-test statistics for the normalized average *SW*_*avg*−*norm*_, *C*_*avg*−*norm*_, and *L*_*avg*−*norm*_ indicies over the five frequency bands.

		**Delta**		**Theta**		**Alpha**		**Beta**		**Gamma**	
		***p*-value**	***t-stat*.**	***p*-value**	***t-stat*.**	***p*-value**	***t-stat*.**	***p*-value**	***t-stat*.**	***p*-value**	***t-stat*.**
*SW* index	Passive	5.60 × 10^−6^	-7.17	4.40 × 10^−5^	-5.78	0.2423	-1.39	0.2423	1.35	0.836	0.21
	active	0.016	-3.08	2.30 × 10^−3^	-4.27	0.309	1.37	0.721	0.362	0.368	-1.08
*C* index	Passive	1.16 × 10^−6^	-6.23	1.76 × 10^−5^	-8.03	0.335	-1.32	0.516	0.839	0.803	-0.252
	active	9.25 × 10^−3^	-3.47	9.25 × 10^−3^	-3.34	0.435	0.965	0.721	0.361	0.403	-1.21
*L* index	Passive	8.75 × 10^−6^	6.94	1.343 × 10^−4^	5.25	0.321	1.17	0.17	-1.72	0.526	-0.647
	active	0.098	2.22	1.15 × 10^−3^	4.58	0.110	-1.96	0.886	0.146	0.840	0.431

Reported *p*-values are the adjusted *p*-values after considering multiple comparisons.

### 3.2. Nodal connectivity properties

**Figure 5:**
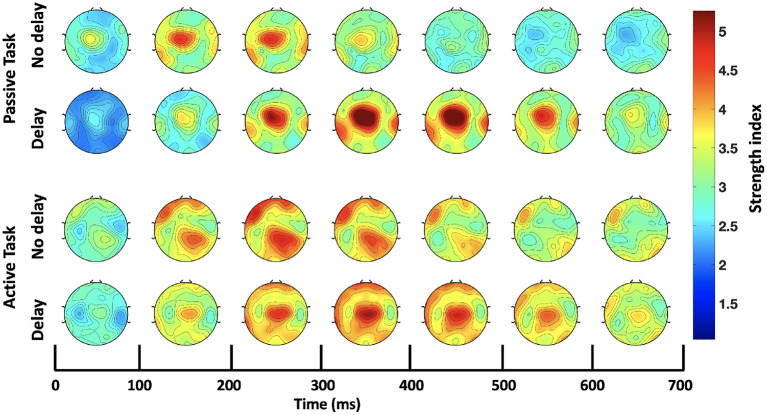
Time-course of the strength index (*S*) topography over the delta-theta bands (1–8 Hz).

**Figure 6:**
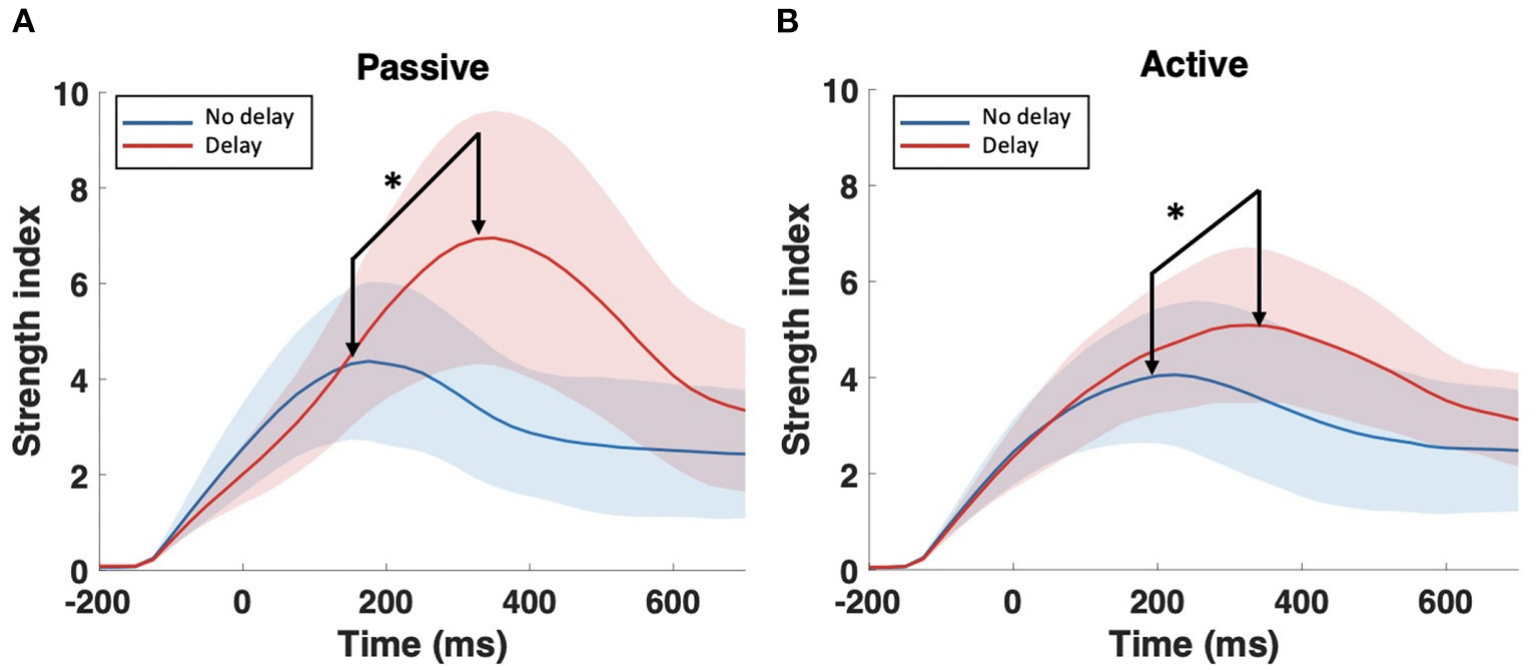
Time-course of the strength index *S* at Cz (1–8 Hz) under the synchronous and delayed conditions for the **(A)** passive task and the **(B)** active task. Arrows point to peaks where statistical tests were performed. *Indicates statistically significant difference.

Consequently, further analysis was focused only on the bands that showed significant differences in the *SW*_*avg*−*norm*_ index, namely, delta (1–4 Hz) and theta (4–8 Hz). The *S* index was computed for each channel (node) over the time-course of the epoch; Figure 5 shows the temporal change of the *S* index topography for the four conditions (PND, PD, AND, and AD). In the passive task, a high localized *S* index is observed in the middle central region centered around Cz. In the presence of delay, the high localized *S* index is temporally shifted and increased in magnitude. In the active task on the other hand, a high *S* index is observed over the frontal and ipsilateral parietal regions under the synchronous condition. In the presence of delay, however, a high localized *S* index is again observed over the middle central region. In all four conditions, the described activation fades out 700 ms after the onset. Since Cz is the center of the activation in the middle central region, the time-course of the *S* index at Cz is extracted and shown in Figure 6. From a temporal perspective, it can be clearly observed that the *S* index peaks later and higher in the presence of delay (PD and AD conditions). The difference is statistically significant and observed over peak values (Passive: *p*-value < 0.05, *t*_*PND*_=[150 ms] and *t*_*PD*_ = [350 ms], paired *t*-test; Active: *p*-value < 0.05, *t*_*AND*_=[225 ms] and *t*_*AD*_ = [350 ms], paired *t*-test).

### 3.3. Inter-regional functional connectivity

**Figure 7:**
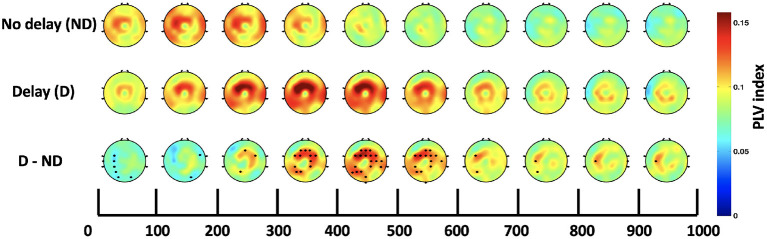
Time course of topography plots of Cz-seeded delta-theta connectivity for the passive task under both conditions and their difference. The highlighted channels in the third row show a significant difference between the delay and no delay conditions.

**Figure 8:**
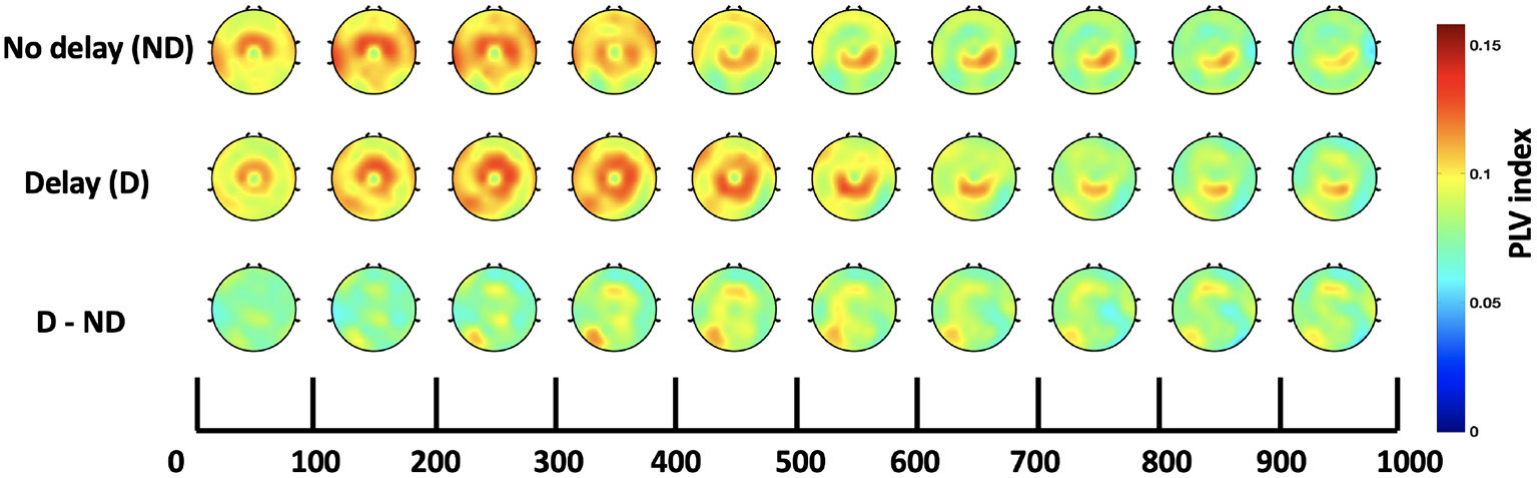
Time course of scalp topographies plots of Cz-seeded delta-theta connectivity for the active task under both conditions and their difference.

Both, the frequency bands and the hub of connectivity were identified through the *SW* and *S* indices, respectively. Connectivity in the delta and theta bands was found to be impacted by haptic delay, and the mid-central region, centered around Cz, was found to be the hub of connectivity. Thus, the Cz-seeded connectivity is plotted in Figures 7, 8 for the passive and active tasks, respectively. Figure 7 shows that there is considerable intra-regional connectivity between Cz and the rest of the middle central channels, particularly connected in PD condition. The difference between PND and PD shows that in addition to the intra-regional connectivity in the middle-central region, there is a significant functional connectivity between Cz and (P1, P3, P5, and CP3) as well as (POz and Oz) located at the contralateral parietal and occipital regions, respectively. Those differences are most pronounced between 400 and 500 ms post the visual collision event. Similar analysis for the active task demonstrates a difference in functional connectivity between Cz and (P3, P5, PO3, PO7 and O1) in the contralateral parieto-occipital region maximally pronounced between 300–400 ms post the onset. However, this difference became insignificant after considering the correction for multiple comparisons.

## 4. Discussion

This study applied a graph theory-driven approach to PLV connectivity patterns derived from EEG data with the objective of identifying distinct global and nodal properties of the neural networks associated with haptic delay during visuo-haptic tasks (passive and active). Our results suggest that the brain exhibits significantly different network characteristics when a haptic delay is introduced. In particular, a significantly increased manifestation of the small-world topology in the theta and delta bands was observed when the haptic delay was introduced (Table 1). An increase in the *SW* index could be attributed to either an increase in the global clustering (neural segregation) or a decrease in the characteristic path length (neural integration). The results in Figures 3B,C and Table 1, show that this increased manifestation in small-worldness is due to both, the significant increase in the clustering coefficient and the decrease in the characteristic path length. In other words, an enhanced small-world topology in the presence of haptic delay indicates increased segregation within the neural clusters and increased integration among the clusters, demonstrating high communication speed between nodes (electrodes) (Bassett and Sporns, 2017). However, the significance test shows that haptic delay had a larger impact on the network properties (*SW*,*C*, and *L*) during the passive task compared to the active task. As reported in self-reporting research (Vogels, 2004), the sensitivity to haptic delay is stronger during passive interactions as compared to active ones. One possible explanation is that during the passive task, participants are perceptually less distracted, making them more receptive to and aware of the delayed haptic feedback.

A delay in an expected haptic stimulus is hypothesized to elicit higher levels of attention when compared to synchronous stimulation. This is, in turn, functionally related to the ability of an individual to detect and respond to rapid and subtle changes in the environment, such as the presence, absence, or delay of an expected stimulus; this ability is known as the psychomotor speed (Bigler, 2012). In light of this hypothesis, the importance of theta band phase synchrony is evident in the literature with respect to cognition (Klimesch, 1999; Axmacher et al., 2006). In particular, theta band clustering and small-worldness were associated with attention (Gootjes et al., 2006), psychomotor speed, and working memory (Douw et al., 2011), which corroborate the current results.

Further analysis of the graph nodal properties in the delta and theta bands revealed high connectivity localized at the middle central region for the PD and AD conditions, as well as the PND condition, see Figure 5. As for the PND condition, the main neural processes involved while performing the task could be more perceptual and less related to motor coordination. A study based on visuo-tactile stimulation (Kanayama et al., 2015) found increased theta phase synchrony at the middle central region (originated at the anterior cingulate) following multisensory (visuo-tactile) stimulation, relative to unisensory (visual) stimulation. However, the pattern of high *S* index at the middle central region in the PND condition is delayed and amplified significantly upon the introduction of the haptic delay, as shown in Figure 6A. The delay could simply relate to deferred multisensory processing upon the late delivery of the haptic feedback, while the significant increase in the *S* index is similar to reported findings indicating a relationship between tasks that involve conflict processing and a subsequent increase in the middle central theta-based connectivity (Wang et al., 2005; Cavanagh et al., 2009, 2010; van Driel et al., 2012).

The AND condition, on the contrary, showed a distinct *S* index pattern, mainly eminent at the frontal and contralateral parietal regions. Previous clinical studies stated that patients with an impairment in central executive functions (i.e., directing attention, maintaining task goals) show reduced fronto-parietal EEG phase synchrony in the theta band (Babiloni et al., 2004); thus, the observed connectivity could imply active employment of central executive functions during the AND condition. However, the connectivity pattern is diverted to the middle central region when the haptic delay is introduced (AD condition). At this stage of understanding, it is tempting to think that the processes related to conflict resolution could be more prominent compared to executive functions generally present in the AND condition.

In addition to the strong intra-regional connectivity in the middle-central regions, significant inter-regional connectivity was also observed. The contralateral parietal and occipital regions were significantly more connected to Cz under the presence of haptic delay for the passive task. Due to the scarcity of research studies that explore the brain functional networks associated with the haptic modality, it is hard to directly relate the current results to previous work. However, a strong speculation relates the observed theta phase synchrony at the parietal region to hippocampal theta activity, pointing to mechanisms of sensorimotor integration (Bland et al., 2007; Watrous et al., 2011). This hypothesis ties well with previous studies wherein theta activity at the parietal cortex plays a major role in supporting body motor movements necessary for goal pursuit or task completion (Guterstam et al., 2015; Lin et al., 2022). By extension, we believe that theta phase synchrony played a significant role in detecting the delay in the haptic modality (middle central region) and communicating the conflict to the parietal cortex for integrating the flow of the incongruent sensory information. Additionally, it is interesting to observe the connectivity between Cz and the occipital electrodes (POz and Oz) indicating a sort of information transfer possibly falling under reactive control processes required to resolve conflict in the involved sensory modalities (motor, visual) (Cooper et al., 2015). In Figure 8, it can be observed that there is higher connectivity between Cz and the contralateral parieto-occipital region (P3, P5, PO3, and PO7) in the AD compared to the AND condition. Despite the difference, it was found insignificant after correcting for the number of channels and the time-bins.

One limitation of this study is that we used the PLV measure to construct the adjacency matrices to evaluate the connectivity between electrode pairs. It is known that the PLV measure is sensitive to the problem of volume conduction, which could result in spurious connectivity (Bruña et al., 2018). However, PLV is one of the standard methods that is used to study connectivity at the scalp level. To address this limitation, we used spatial filtering (CSD method), which is known to attenuate the volume conduction effect (Srinivasan et al., 2006). An interesting future direction would be to study the effects of changing the type of haptic feedback (i.e., vibrotactile or kinesthetic), the amount of haptic delay, and the variation in delay (i.e., jitter) on the functional networks involved in processing haptic delay.

## Data availability statement

The raw data supporting the conclusions of this article will be made available by the authors, without undue reservation.

## Ethics statement

The studies involving human participants were reviewed and approved by New York University Abu Dhabi Institutional Review Board (IRB: #HRPP-2019-120). The patients/participants provided their written informed consent to participate in this study.

## Author contributions

ME and HA conceived the study. HA and WP designed the experimental protocol. HA conducted the experiment and analyzed the results. WP and ME reviewed the results. All authors contributed intellectually in writing and revising the manuscript.

## Funding

This research was funded by the New York University Abu Dhabi PhD Fellowship Program. This work is also supported in part by the NYUAD Center for Artificial Intelligence and Robotics, funded by Tamkeen under the Research Institute Award CG010.

## Conflict of interest

The authors declare that the research was conducted in the absence of any commercial or financial relationships that could be construed as a potential conflict of interest.

## Publisher's note

All claims expressed in this article are solely those of the authors and do not necessarily represent those of their affiliated organizations, or those of the publisher, the editors and the reviewers. Any product that may be evaluated in this article, or claim that may be made by its manufacturer, is not guaranteed or endorsed by the publisher.
